# Conceptualising and Teaching Biomedical Uncertainty to Medical Students: an Exploratory Qualitative Study

**DOI:** 10.1007/s40670-021-01481-x

**Published:** 2022-01-17

**Authors:** Eva Lukšaitė, Rosemary A. Fricker, Robert K. McKinley, Lisa Dikomitis

**Affiliations:** School of Medicine, David Weatherall Building, Keele University, Keele, ST5 5BG Staffordshire UK

**Keywords:** Biomedicine, Interviews, Medical curriculum, Situated knowledge, Qualitative research

## Abstract

**Introduction:**

Certainty/uncertainty in medicine is a topic of popular debate. This study aims to understand how biomedical uncertainty is conceptualised by academic medical educators and how it is taught in a medical school in the UK.

**Methods:**

This is an exploratory qualitative study grounded in ethnographic principles. This study is based on 10 observations of teaching sessions and seven semi-structured qualitative interviews with medical educators from various biomedical disciplines in a UK medical school. The data set was analysed via a thematic analysis.

**Results:**

Four main themes were identified after analysis: (1) ubiquity of biomedical uncertainty, (2) constraints to teaching biomedical uncertainty, (3) the ‘medic filter’ and (4) fluid distinction: core versus additional knowledge. While medical educators had differing understandings of how biomedical uncertainty is articulated in their disciplines, its presence was ubiquitous. This ubiquity did not translate into teaching due to time constraints and assessment strategies. The ‘medic filter’ emerged as a strategy that educators employed to decide what to include in their teaching. They made distinctions between core and additional knowledge which were defined in varied ways across disciplines. Additional knowledge often encapsulated biomedical uncertainty.

**Discussion:**

Even though the perspective that knowledge is socially constructed is not novel in medical education, it is neither universally valued nor universally applied. Moving beyond situativity theories and into broader debates in social sciences provides new opportunities to discuss the nature of scientific knowledge in medical education. We invite a move away from situated learning to situated knowledge.

## Introduction

‘As a clinician, […] I have seen paradigms change beyond recognition and actions which were considered tantamount to attempted murder becoming best practice within half a professional lifetime’ R. K. McKinley, research team member

‘In medicine today, uncertainty is generally suppressed and ignored, consciously and subconsciously’ [1]

The key rationale for this study was the dissonance between how widely biomedical uncertainty has been discussed in medical education scholarship [1–10] and how little of these discussions have made their way into the corridors of the medical school where this research team is based. There is a disjunction between physicians’ experience of uncertainty in knowledge and clinical practice and a culture of medicine with its ‘deep-rooted unwillingness to acknowledge and embrace it’ [1].

The concept of biomedical uncertainty has been investigated in medical schools [2], clinical practice [3, 4], case presentations [5], patients’ construction of identities [6], and as a multifaceted concept in health care [6]. While Fox [8–10] argues that medical education teaches students to deal with uncertainty, Atkinson [11] suggests that medical students are educated for certainty and that medical knowledge is neither presented by teachers nor accepted by students as problematic.

Fox [8–10] conceptualises three main types of uncertainty in medical school: (1) uncertainties originating in the impossibility of commanding all knowledge in a constantly changing medical field, (2) uncertainties arising from gaps in medical knowledge and (3) uncertainties based in the struggle to distinguish between medical students’ personal ineptitude and the limits of medicine. Even though a lot of research investigates different manifestations of uncertainty in medicine and mechanisms to manage it, there is little conceptual clarity on what uncertainty really means [7]. The uncertainty of knowledge has not been investigated deeply in medical education, even though scientific uncertainty is an object of study in other disciplines [12].

Many authors call for the development of clinicians’ ‘tolerance of uncertainty’ as key to humanistic health care [1, 4, 13, 14]. Our study builds on this certainty–uncertainty debate and focuses on uncertainty related to *biomedical* learning content in an integrated, undergraduate curriculum. Biomedical uncertainty in our study covers historical changes in clinical practices, differences in medical protocols across the world, gaps in current understandings of pathophysiology of certain conditions and conflicting evidence of fast-moving science [15, 16]. We suggest that learning to handle uncertainty in clinical settings begins with learning to handle uncertain knowledge in the foundational medical sciences curriculum. We aim to contribute to conceptualisations of uncertainty and provide some recommendations which may support the management of uncertainty in clinical practice.

Philosophers of science have long argued that scientific claims reflect the historical and sociocultural context in which those claims were made [17, 18]. Within this field, scientific facts are never more than arbitrary agreements on the basis of whatever is considered to be ‘evidence’ at any given time and in any given discipline. Feminist scholars [17, 19–24] made significant contributions to the construction of scientific knowledge debate by conceptualising the idea of ‘situated knowledge’. Feminist theory presents a challenge to positivist notions of objectivity and the possibility of a universal knowledge by critiquing the idea that researchers are neutral observers of reality and that teachers are neutral translators of knowledge. Instead, knowledge cannot be separated from those producing or delivering it and their social positioning must be accounted for [24]. The idea of ‘situated knowledge’ [19] critiques the figure of the modern scientist who is assumed to be free from sociocultural ties and capable of knowing reality without bias.

Social constructivism is not a novel perspective in medical education. One of its manifestations is situativity theory [25, 26] which encompasses various frameworks that argue that knowledge and learning are situated in experience. Originating in cognitive psychology and widely applied in medical education, situativity theory resonates with the idea of situated knowledge within feminist scholarship [19]. Experiences that provide a context for knowledge and learning include culture, physical environment and interactions amongst learners [25]. Situativity theory is more preoccupied with the process of learning rather than the nature of knowledge, a direction that the idea of situated knowledge takes. This article builds on the existing work on uncertainty from a situativity theory perspective and focuses on the situatedness of knowledge rather than learning.

We conducted empirical exploratory research within one medical curriculum, which itself is a product of sociocultural, political and economic forces [27, 28]. We aim to elicit conversations about the nature of knowledge, especially in the light of ongoing initiatives to decolonize medical curricula [29]. Insights from social sciences and feminist theory, which have been largely absent in medical education [30], can help in rethinking the absoluteness of biomedical knowledge and what constitutes ‘evidence’ [31, 32].

## Methods

### Study Design

We used a qualitative approach underpinned by principles of ethnographic research [33] which is, by definition, exploratory [34]. Exploratory research projects offer insights that can inform our knowledge and suggest future directions without being guided by very precise and limiting hypothesis or a pre-determined gap in literature. A qualitative approach allows respondents’ experiences to emerge freely without a pre-determined set of questions. It allows for the research problem to be defined by respondents and emerge from empirical data. An observation of teaching sessions facilitates a collection of data in ‘natural’ settings.

### Field Site

The study was carried out in a medium-sized medical school in the UK with an intake of approximately 2/3 post-secondary and 1/3 post-degree education and over 90% from the UK and Europe. The medical curriculum is delivered over 5 years by academic and clinical medical educators who are involved in a broad range of teaching subjects and formats. The medical school delivers an integrated spiral curriculum structured around problem-based learning (PBL), small-group teaching and student-directed learning. The first 2 years of the curriculum are delivered through thematically structured units where different disciplines are taught together in an integrated manner with a special attention to social aspects of medicine. The first 2 years of the medical degree are mainly classroom-based and the remaining 3 years are in a broad range of clinical settings.

### Recruitment Procedures, Consent and Ethics

The study was reviewed and received full ethical approval from Keele University Ethics Review Panel (ERP Ref: ERP2377). All colleagues who were delivering lectures and practical classes to Year 1 (Y1) and Year 2 (Y2) students during the time of the study were approached about being observed for the study. Some colleagues stated that their sessions would not contain any examples of uncertainty; some did not agree to be observed for undisclosed reasons. Convenience sampling was employed for interviews and colleagues from different disciplines were approached informally about participating in the interviews. Before each observation and at the beginning of each interview, the project aims were explained alongside the discussion on anonymity, confidentiality and the withdrawal process. Respondents were informed that they were free to withdraw from the study at any point until the final drafting of results. Informed consent was obtained prior to observed teaching sessions and prior to the start of the interview. All data in this article have been pseudonymised. The draft of this article was shared with all respondents who agreed that the level of pseudonymisation was sufficient.

### Data Collection

Data were collected via observations of teaching sessions and semi-structured qualitative interviews with academic medical educators (Table 1). Data collection was carried out by a medical anthropologist (EL) in 2018. During observations, EL sat in the middle of the lecture theatre or in the corner in the laboratory, noting features which she considered pertinent to the study and made detailed field notes of her observations.

**Table 1:** Characteristics of respondents

**Respondent identifier**	**Gender**	**Discipline**	**Years of teaching at the medical school**
R1	M	Microbiology, infectious diseases	13
R2	F	Anatomy, histology	1
R3	F	Immunology	6
R4	M	Physiology	8
R5	F	Muscle biology	1
R6	M	Pharmacology, physiology	15
R7	M	Cell biology	4

To elicit discipline-specific conceptualisations, biomedical uncertainty was explained as change, contradictions and the unknown in biomedical sciences. Research participants were then asked to provide examples of such topics from their own disciplines. Based on literature and one pilot interview, researchers developed an interview guide. Interviews were purposefully limited to research participants’ experience of teaching Y1 and Y2 students. Interviews lasted between 45 and 90 min and were recorded and transcribed with permission of the interviewees.

### Data Analysis

Thematic analysis was used to analyse the collected data. We identified, analysed and interpreted themes that we saw emerging in field notes and interview transcripts inductively, without a pre-existing framework. Transcripts were read and discussed in team meetings. We acknowledged an active role that we as researchers played in the process of constructing themes [35] through an active reading of the data and relating it to our experiences as educators in the medical school. Themes’ importance was determined by their relevance to the research question and potential to provide theoretical insight rather than by frequency. We used the following procedure for theme development: we read and re-read the whole dataset of interview transcripts and fieldnotes, manually coded the dataset, then generated initial themes, reviewed them and, finally, defined and named themes [35].

### Reflexivity

The experience of the lead researcher (EL) as a medical anthropologist working in a medical school has directed the formulation of the research questions and methods. The project’s exploratory nature was driven by EL’s anthropological training and her experience of how biomedical knowledge is often discussed by students as certain and universal even though paradigm shifts in knowledge and clinical practice are abundant. The research team is comprised of four medical educators with interdisciplinary backgrounds. EL is a medical anthropologist, RF is a neuroscientist, RM is a general practitioner and LD is a social and medical anthropologist. At the time of data collection and analysis, all authors worked at the medical school where research was carried out. Conversations within this interdisciplinary team surrounding study design, data collection and analysis allowed discipline-specific biases to be reduced.

## Results

Seven semi-structured qualitative interviews were conducted, and 10 teaching sessions (lectures and laboratory-based practicals) were observed. The core teaching team was comprised of 24 bioscience educators and interviewed participants represented most bioscience disciplines (Table 1). Data saturation was achieved as no new themes emerged towards the end of the study [36]. After thematic analysis of collected data, four main themes were identified: (1) ubiquity of biomedical uncertainty, (2) constraints to teaching biomedical uncertainty, (3) the ‘medic filter’ and (4) the fluid distinction: core versus additional knowledge.

### Ubiquity of Biomedical Uncertainty

Most observed sessions included references to biomedical uncertainty (Table 2). Even though references to uncertainty were fewer than the content that was considered to be established scientific knowledge, uncertainties were inextricably intertwined with established knowledge. They were mostly presented as a matter of fact, in passing and were rarely discussed in-depth. During interviews, respondents provided many more examples of biomedical uncertainty from their disciplines: pathogenesis of Ebola virus, mechanism of action of paracetamol and antidepressants, pathophysiology of Crohn’s disease and depression, the process of haematopoiesis, understanding of the microbiome and neurodegeneration. All interviewees recognised that uncertainty plays a significant role in their disciplines. Biomedical uncertainty was mostly articulated as change in biomedical research findings, theories and practices. The perceived significance of this change can best be illustrated with the words of one respondent:

‘What we refer to as [HIV and AIDS] was only fully described in 1981. And in the space of the time between us realising it was something, the development of the AIDS pandemic and the development of antiretroviral therapies to manage it, only 25 years had elapsed. And in the space of not even somebody’s clinical lifetime, we have changed the way in which we look at the world, the way we identify a condition, the way that we manage it.’ (R1)

**Table 2:** Examples of biomedical uncertainty in observed teaching sessions

**Session title and year**	**Example of biomedical uncertainty**
Renal physiology, Y1	The paradigm shift in the understanding of the role of the kidney in plasma pH regulation
Genes and the basis of genetic medicine, Y1	The still-evolving understanding of a ‘gene’ from a single strand of DNA coding a single protein to a dispersed entity with multiple functions, which is still not fully understood
Pain physiology and pharmacology, Y2	We still do not know why the body changes from physiological to pathological pain
Human reproduction, Y1	The evidence base for the management of infertility is not as strong as one might think
Neurobiology of pain, Y2	Functions of nociceptors are currently poorly understood

Change is a fundamental requirement of the scientific method and its prevalence in these definitions is not surprising. However, interviewees’ conceptualisations of uncertainty, its pace and source differed significantly between disciplines (Table 3). A microbiologist argued that microbiology contains a large amount of uncertainty due to the constant mutation of organisms causing disease. Immunology was presented as one of the fastest-moving disciplines with biomedical uncertainty as its integral part. In anatomy, uncertainty was articulated as anatomical variations and reclassification of organs and structures. The place for uncertainty was also acknowledged in physiology, even though to a smaller degree.

**Table 3:** Interview extracts of discipline-specific conceptualisations of biomedical uncertainty

**Discipline**	**Discipline-specific conceptualisation of biomedical uncertainty**
Microbiology	‘Prescribing guidelines change in response to development of reduced sensitivity to drugs that we would use previously. That occurs, as I said, in a temporal fashion through time, but also within geographical areas, within healthcare trusts, those things change, and they can change quickly. So whereas prescribing guidelines for certain drugs may take years to change, those for antimicrobials can sometimes change in a matter of months.’ (R1)
Immunology	‘There are certain fundamental core concepts in immunology that we've had nailed down for several years, but otherwise, you could go month to month and new things are discovered […]. When I learned it how many years ago, I was taught about two types of T cells. Now when I started teaching here (5 years ago), I was talking to them about three types. Now we're up to four, five, six different types of T cells. Next year who knows how many different things there might be. It is an area that’s constantly evolving, constantly being researched.’ (R3)
Anatomy I	‘You can look for variations in the configurations of arteries or of nerves, and so what’s happened in anatomy is that a lot of this variation and uncertainty has actually been kind of condensed into we expect this, this is our normal anatomy. But I think it’s very unlikely that anyone in the world has the whole normal anatomy, because there are so many variations to muscles and arteries and nerves and brains that I don’t think anyone has, from beginning to end, what the textbook says they have.’ (R2)
Anatomy II	‘You’ll see these things occasionally where suddenly people discover a new organ and this is made a lot of in the newspapers, so most recently it was the interstitium. […] So that has recently been designated by someone an organ. Now if that will remain an organ or if that were shouted down, that’s a different thing. But we’ve known for a substantial period of time that there was this interstitial fluid, but then the designation as an organ is fairly recent. And another example from a few years before that, we’ve discovered a new organ and what it was it was the mesentery. […] And this was recently designated an organ. And all the news stories sounded very much like humans have just discovered this thing. And it’s like well, we’ve always known that’s there, and it’s always had a name, it’s just now we’ve decided it’s an organ.’ (R2)
Physiology	‘We know how a heart attack works, but how it’s triggered is different, so some things, the pathology stays the same, but the mechanism leading to that will change. There are things where it’s a bit of both, that basically some parts of these things are static. We know the answer or we’re pretty certain we know the answer, we just don't know how that happens, and it depends on the level of detail that we’re going into. (R4)’

### Constraints to Teaching Biomedical Uncertainty

Acknowledging that biomedical uncertainty plays important roles in each discipline did not always translate into teaching it. Research participants recognised various constraints which prevented them from including such topics into their teaching.

The limited time given to each discipline in an integrated medical curriculum was seen as key structural constraint to including uncertain knowledge:

‘Unfortunately, I don’t have time in a lot of the lectures to really […] talk about [uncertainty], but I at least use it as a cue for students to understand that this is not a complete given, this is an area of change and this is something that they may want to look into further.’ (R4)

The assessment system was identified as another structural constraint to teaching uncertainty. Respondents discussed the need to be highly selective in what knowledge was tested in examinations. Types of assessments and exam questions were also seen as limiting the possibility of testing uncertainties. Respondents supposed that due to students’ characteristic of being exam-focussed and the fact that we rarely assess on uncertainty, students pay less attention to subjects containing explicitly articulated uncertainty.

### The ‘Medic Filter’

Respondents did not perceive exclusion of uncertainty from their teaching to be a significant shortcoming. Such judgement was based on the ‘medic filter’ (R5). The idiom ‘medic filter’ refers to the process of making decisions concerning which biomedical knowledge is relevant for clinical practice. The distinction between a medical student and a science student and their different necessary knowledge bases were made by most respondents. When discussing the application of the ‘medic filter’ to rheumatoid arthritis, one respondent narrated:

‘You need to know that it’s these cells and these inflammatory molecules because they are the ones that we clinically and medically target. Whether they’re macrophages and M1, M2 A B C or D doesn't matter necessarily to a clinician unless they're going to go into rheumatoid arthritis research later.’ (R3)

Many respondents acknowledged that applying the ‘medic filter’ presents its own challenges:

‘Our understanding of how haematopoiesis works is not very well understood, really, and is extremely complex. To the level that the medical students need to understand it, to package it down into one lecture and a practical is very difficult. It's a sort of thing that when I would do biology, it was part of, say, an eight-week unit, whereas I do one lecture. Trying to balance that area in terms of the content being relevant to the medical students as medics and not confusing them too much with the content but enough that they understand it, so that’s the area I find most difficult to teach.’ (R3)

### The Fluid Distinction: Core Versus Additional Knowledge

While applying the ‘medic filter’, research participants made a distinction between core and additional knowledge. Participants described core knowledge situated somewhere between ‘fairly well-established’ and ‘facts’, whereas additional knowledge included theories, fast-moving science, subtleties and reclassifications. An immunologist said:

‘I really try to get this idea across to them that there are core concepts and processes, and the specifics of things that do potentially change, but just because we had eight receptors last year and this year we have 12 receptors, they are still receptors that recognise something that instruct the immune system.’ (R3)

Medical educators divided their disciplinary knowledge into core and additional. The proportion of core and additional knowledge taught varied greatly between disciplines. Whereas uncertainty took a more prominent role in immunology and microbiology, it played a much smaller role in the teaching of anatomy, cell biology and physiology. An anatomist said:

‘A lot of it is context in my teaching. And I don’t teach it too much. I try and hint that it’s there and that science isn’t finished because I think that there’s a real perception [that] surely we know everything. No, not about anything frankly. We don’t know everything about anything.’ (R2)

## Discussion

This study demonstrates that uncertainties stemming from gaps and limitations of knowledge, as conceptualised by Fox [8–10], are ubiquitous within most bioscience disciplines taught at the medical school. These uncertainties range from a recent reclassification of the interstitium as an organ to a paradigm shift in the understanding of the role of the kidney in plasma pH regulation. Even though respondents indicated that scientific change is a fundamental part of the scientific method and of various bioscience disciplines, students are taught only a small degree of uncertainty. This is influenced by the perceived constraints of the medical curriculum. The ‘medic filter’ emerges as a strategy to make distinctions between core and additional knowledge.

The study findings and the application of the ‘medic filter’ point to the fluid, creative and fragmented nature of biomedical knowledge that is taught to medical students. It is influenced by structural factors, such as time constraints in a medical curriculum and the assessment strategy, and individual factors, such as educators’ decisions regarding the session content.

This study has implications for the understanding of how biomedical knowledge is taught in a specific context. It is not only the learning that is situated as conceptualised by situativity theory [25, 26]; biomedical knowledge itself is also situated. Medical educators make specific individual and structure-informed decisions on the session content and, thus, produce a particular way in which medical students are taught to understand biological processes. The simplification of complex phenomena through the ‘medic filter’ implies that interpretation is an integral part of this process. There is no neutral position from which to (re)present ‘scientific knowledge’. Knowledge itself is always constructed, constrained and situated.

This study makes several contributions. Firstly, a theoretically and empirically grounded approach to knowledge construction aims to elicit debates on the nature of scientific knowledge within medical education and invite a move away from situated learning to situated knowledge. Biomedical knowledge is rarely problematised within medical sciences. The knowledge translation metaphor — where knowledge is seen as objective, context-free and separable from those who produce and teach it — still dominates medicine [37]. Social science research investigating the social construction of scientific knowledge demonstrates that such knowledge is not an objective, context- and value-free content but, instead, is a product of sociocultural, historical and power relations [38–42].

Secondly, this study empirically investigates the feminist concept of situated knowledge [19] within a medical curriculum and demonstrates how biomedical knowledge is situated and influenced by structural and individual factors. This approach to knowledge destabilises the dominant narrative of a teacher as a possessor of universal knowledge who transmits knowledge in an unproblematic way [24]. The feminist approach has great potential to reconceptualise how and what is taught within a medical curriculum. By outlining how educators make decisions about the content that they cover or skip, this study demonstrates how situated knowledge manifests in a current medical curriculum.

Thirdly, this study expands our understanding of uncertainty in medical education by demonstrating that uncertainty is often excluded from the session content due to time constraints and assessment strategies. This exclusion is often justified by educators through the supposed irrelevance of uncertainty to clinical practice and by deeming uncertain knowledge additional instead of core. This view contradicts scholarly accounts and practitioners’ experiences that uncertainty is a fundamental dimension of clinical medicine [1–5]. We offer some practical recommendations to address the constraints of time and assessments and to reconsider its relevance.

While medical students are just starting to develop strategies to simultaneously uphold the belief in biomedical certainty while comprehending its limits, this fluidity is often fostered as an integral part of the practicing physicians’ worldview through lifelong learning. A continuous, collaborative and active lifelong learning is acknowledged as an essential competency that clinicians must possess to keep up to date with ongoing developments in medicine [43].

### Practical Recommendations

1. To appreciate the ubiquity and relevance of uncertainty, introduce sessions on the social construction of knowledge, the scientific method, history of science, philosophical underpinnings of positivism and the construction of ‘evidence’.
2. Discuss the ubiquity and relevance of uncertainty in discussion-based sessions to develop skills to recognise it and incorporate it in future practice.
3. Consistently mention during the existing sessions if other conceptualisations/protocols were hegemonic before the introduction of current guidelines. Succinctly contextualising existing protocols may help develop students’ understandings of uncertainty in time-constrained curricula.
4. Modify the structure of assessments to incorporate possibilities of multiple answers, viewpoints and interpretations.

### Strengths and Limitations of the Study

The strengths of the study include an interdisciplinary team and the employment of qualitative research methods which ensured that respondents’ voices emerged freely. Data analysis was conducted by four analysts allowing different interpretations of data to be discussed. The limitations of the study include a possible occurrence of the Hawthorne effect [44], the focus on one integrated medical curriculum in the UK which makes generalisability of study findings difficult and the exclusive focus on academic medical educators’ experiences.

### Future Research Directions

Future research projects could include voices of other stakeholders, such as medical students, compare several medical schools with different characteristics or explore differences between biomedical knowledge as taught in different medical schools and clinical contexts.

## Data Availability

The datasets generated and analysed during the current study are not publicly available due to restrictions imposed by the ethics review process.
